# Environmental niche and functional role similarity between invasive and native palms in the Atlantic Forest

**DOI:** 10.1007/s10530-020-02400-8

**Published:** 2020-11-13

**Authors:** Carolina Bello, Ana Laura P. Cintra, Elisa Barreto, Maurício Humberto Vancine, Thadeu Sobral-Souza, Catherine H. Graham, Mauro Galetti

**Affiliations:** 1 Departamento de Biodiversidade, Instituto de Biociências, Universidade Estadual Paulista (UNESP), 13506-900 Rio Claro, São Paulo, Brazil; 2 Swiss Federal Research Institute WSL, Zürcherstrasse 111, 8903 Birmensdorf, Switzerland; 3 Programa de Pós-graduação em Ecologia e Evolução, Universidade Federal de Goiás, Goiânia, Brazil; 4 Departamento de Botânica e Ecologia, Universidade Federal de Mato Grosso (UFMT), Campus Cuiabá, Mato Grosso, Brazil; 5 Department of Biology, University of Miami, Coral Gables, FL USA

**Keywords:** *Euterpe edulis*, *Archontophoenix cunninghamiana*, Plant-animal interactions, Niche overlap, Climate change, Functional role, Biological invasion

## Abstract

Invasive species can significantly affect native species when their niches are similar. Ecological and morphological similarities between the invasive Australian palm, *Archontophoenix cunninghamiana,* and the native palm from the Brazilian Atlantic Forest, *Euterpe edulis*, suggest that they have similar environmental requirements and functional roles (i.e., the function a species performs in an ecosystem). This similarity raises concerns about how the invasive palm could impact the native species in the present and future. We used spatial (species occurrences) and ecological information (frugivory events) to characterize the environmental niche and functional role of the two palms and assess their overlap. In addition, we predicted the potential area of occurrence of each palm within the Brazilian Atlantic Forest under current and future climate conditions.We estimated the environmental conditions used by the invasive plant based on its native distribution only, and based on all areas where the species is able to establish across the globe. We found that the environmental niches of the two palm species overlap up to 39%, which corresponds to 50% of the current geographic distribution of E. edulis in the Atlantic Forest. In the areas where the two species potentially co-occur, the impact of the invasive species on the native should be influenced by the invasive species interactions with frugivores. We found that the frugivory functional role of the two palms was similar (84% overlap) which suggest that *A. cunninghamiana* might disrupt the seed dispersal of the native palm. However, co-occurrence between the palms may decline with future climate change, as the potentially environmental suitable area for the invasive palm is predicted to decline by 10% to 55%. Evaluating the similarity in both the environmental niche, of the native and global extent, and the functional role of native and invasive plants provides a detailed understanding of the potential impact of invasive species on native species now and in the future.

**Electronic supplementary material:**

The online version of this article (10.1007/s10530-020-02400-8) contains supplementary material, which is available to authorized users.

## Introduction

Biological invasion causes economic and ecological losses and is a key threat to biodiversity worldwide (McGeoch et al. 2006; Pimentel et al. 2005; Sala et al. 2000). Often, invaders alter biodiversity patterns by colonizing new areas with a suitable environment where they exploit empty niches, have lower susceptibility to natural enemies, and often compete with native species (Catford et al. 2009). The impact of invasive species on native ones has predominantly been evaluated based on overlap in the species environmental requirements, while the effect on the functional role of native species is often ignored (Rosado et al. 2016). Here, we address this gap by jointly exploring the similarity in the environmental requirements and functional roles of an invasive and a native palm species in the Atlantic Forest of Brazil.

Potential areas of invasion are generally identified based on the environmental requirements that determine the occurrence of a given species and is often quantified at a broad spatial scale (Jiménez-Valverde et al. 2011a, b; Peterson et al. 2008). However, the occurrence of a species at local scales also depends on density-dependent processes, such as the functional role that species perform through their biotic interactions (Chase and Leibold 2003; Rosado et al. 2016; Soberón 2007). An invasive species, that has a similar functional role as a native species in the invaded area, can alter the mutualistic or antagonistic interactions of the native species (Mooney and Cleland 2001). Invaders can monopolize pollinators and seed dispersers, leading to reduced reproductive and seed dispersal rates of native plants (Brown and Hopkins 2002; Greenberg and Walter 2010; Traveset and Richardson 2006; Vila and D’Antonio 1998). Therefore, the degree of similarity between the functional role of an invasive and native species enables us to anticipate more substantial impacts than those solely based on the environmental niche. The similarity of functional roles between two species can be estimated based on the similarity of the functional traits of interacting species (Dehling and Stouffer 2018). An overlap in functional traits of these interacting species indicates that the species have similar functional roles in a community (Dehling and Stouffer 2018). In the case of seed dispersal, the functional roles of the plant species can be determined by the functional traits of their seed dispersers (Dehling et al. 2016; Elton 1927).

*Archontophoenix cunninghamiana* H.Wendl. & Drude is a native palm of the tropical rainforests of Australia that has been introduced worldwide for ornamental and food production purposes (Dowe 2009; Lorenzi et al. 1996). In the Atlantic Forest of Brazil, the invasion of *A. cunninghamiana* was reported more than a decade ago and has expanded extensively (Dislich et al. 2002; Matos and Pivello 2009). This rate of expansion can impact an ecologically similar endemic native species, *Euterpe edulis* Mart., because both species have similar growing habit, infructescence structure, and fruit and seed size and color (Christianini 2006). In addition, the invasive palm has faster growth and germination rates compared to *E. edulis* (Mengardo and Pivello 2014). The impacts of *A. cunninghamiana* on *E. edulis* can generate important secondary effects on other species because *E. edulis* is a keystone food resource in the Atlantic Forest (Galetti and Aleixo 1998). *Euterpe edulis* occurs in high abundance in natural conditions and provides food for different taxa. Its fruits are consumed by more than 50 bird species, from thrushes to toucans, and more than a dozen mammal species (Galetti et al. 2013, 1999). Moreover, the loss of large seed dispersers (i.e., defaunation) has resulted in phenotypic and genetic homogenization of local *E. edulis* populations (Carvalho et al. 2016; Galetti et al. 2013). *Euterpe edulis* now faces risk of extinction as a result of deforestation and illegal extraction for heart palm consumption (Galetti and Fernandez 1998).

The Atlantic Forest of Brazil is a Neotropical hotspot of biodiversity that ranges from approximately 5°–33° in latitude South across the eastern coast of Brazil reaching continental areas in northeastern Argentina and southeastern Paraguay (Morellato and Haddad 2000). These areas are extremely heterogeneous in terms of climate, soil and altitude. Consequently, the Atlantic Forest has several types of forests, including evergreen, semi-deciduous, deciduous, mixed temperate (locally known as Araucaria forests), white-sand (Restingas and Mussunungas), open canopy (Brejos de altitude), alluvial, cloud and swamp forests (Oliveira-Filho and Fontes 2000). The Atlantic Forest has been impacted by the synergistic effects of climate change and other components of global change, such as habitat fragmentation and biological invasions (Bellard et al. 2014). The extreme level of deforestation of the Atlantic Forest, where just 12% of the forest remains, mostly in small fragments (Ribeiro et al. 2009), and the high rate of *A. cunninghamiana* propagations, suggest that urgent actions are needed to detect the possible ecological effects of the *A. cunninghamiana* invasion under current and climate change scenarios.

Currently, species distribution models (SDMs) are one of the main statistical approaches to identify potential areas of species invasion under current and future climatic scenarios (Bradley et al. 2010; Walther et al. 2009). SDMs are based on correlation between known species occurrences and environmental conditions to infer indirectly their environmental niches. These relationships are then plotted in geographical space (Peterson 2011). SDMs also are an important tool for predicting potential species invasion risk at broad-scales (Jiménez-Valverde et al. 2011a, b). At local scales, the strength of *A*. *cunninghamiana* invasion will also be influenced by the potential changes on native species biotic interactions. Thus, we explored the similarity in environmental requirements and functional roles between *A. cunninghamiana* and *E. edulis*, and identified possible areas where *A. cunninghamiana* is predicted to spread and influence the functional role of *E. edulis*. By using SDMs and projecting the potential distribution of both species in the Atlantic Forest, we identified areas of potential invasion in the Atlantic Forest under current and future climatic conditions. By exploring the functional traits of the frugivores that interact with the two palms in the Atlantic Forest, we found that *A. cunninghamiana* has the potential to influence the functional role of *E. edulis* by disrupting its frugivory interactions.

## Methods and study site

*Archontophoenix cunninghamiana,* known as Bangalow or King palm, is a monopodial shade tolerant palm tree that grows faster in full sun. Because it is a highly prolific seeder, and tolerant to different soil conditions, it has successfully invaded areas of Australia, New Zealand, Hawaii and Brazil (Atlas of Living Australia 2017). This species has an extended fruiting period, flowering and fruiting the entire year in some areas, and produces small red drupes (fruit diameter of 9.8 ± 0.97 mm) that are very attractive to birds (Mengardo and Pivello 2012). Seeds can germinate quickly (1 to 3 months) and after germination, seedlings can persist for years, forming a seedling bank in the forest, waiting for appropriate conditions to germinate (Williams 2008). In Brazil, *A. cunninghamiana* is well adapted to the subtropics, where environmental conditions are similar to those of its native localities (Lorenzi et al. 2004). Besides, it has been widely used for ornamental purposes and palm heart production (Lorenzi et al. 1996).

*Euterpe edulis* is an endemic palm tree from the Brazilian Atlantic Forest, popularly known as Juçara palm. It occurs from south of Bahia to Rio Grande do Sul states (Jarenkow and Waechter 2001; Reis et al. 2000). *Euterpe edulis* is well adapted to low light conditions. In native conditions, *E. edulis* was widely and abundantly distributed in different Brazilian Atlantic Forest formations (Reis et al. 2000). Although there is local variation, fruiting of *E. edulis* begins in May, and fruit ripening occurs until November. Each palm individual produces one to five infructescences, containing more than 1300 fruits per bunch. The fruit is globose drupe (fruit diameter ~ 11.98 ± 1.15 mm), with a thin black epicarp and a carbohydrate-rich mesocarp (pulp) enclosing a single seed.

### Species occurrence and climatic data

We used two occurrence data sets for *A. cunninghamiana*. First, we considered only records from its native area (native area). Second, we considered occurrences from across the globe (native and invaded area). We obtained 3976 occurrences from two online databases: The Atlas of Living Australia (https://www.ala.org.au/about-ala/) and Global Biodiversity Information Facility (GBIF; GBIF.org 2020). We cleaned the data by filtering for occurrences between 1970 to 2020, removing sampling bias (with CoordinateCleaner R package; Zizka et al. 2019), and using spatial disaggregation with 2.5 arc-minutes [with ecospat R package; Broennimann et al. 2015]. After cleaning, we had 159 occurrences in the native range and 483 in the global range. For *E. edulis*, we compiled 248 records from speciesLink (http://splink.cria.org.br/), GBIF and a database from the Laboratory of Conservation Biology (https://ib.rc.unesp.br/index.php#!/departamentos/ecologia/labic/home/). We disregarded occurrences outside Australia and Brazil because mostly these correspond to individuals that were planted or are in botanical gardens where the environmental conditions are manipulated. We reduced the spatial aggregation and environmental biases of occurrence points by using a 10 km spatial rarefaction in SDMtoolbox to prevent model overfit to areas with high density of occurrence (Boria et al. 2014; Boucher-Lalonde and Currie 2016; Brown 2014) (Table S1).

To characterize the environmental niche of each palm species and to perform species distribution modelling, we used 19 bioclimatic variables of current and future climate scenarios (global circulation model GCM: ACCESS1-0, rpc45 and rcp85 for 2050 and 2070) at 2.5 min resolution (approximately ~ 5 km^2^ at the Equator) from WorldClim v.1.4 (Hijmans et al. 2005). These variables have been widely used for modeling the distribution of organisms worldwide and represent biologically significant variables (Porfirio et al. 2014). We sampled the climatic conditions in the areas of occurrence of the two palm trees.

### Overlap in the environmental niche

We explored the similarity in the environmental niches of the two palms using Schoener’s *D* niche overlap metric, which applies kernel smoothers to densities of species occurrence in a gridded environmental space (Warren et al. 2008). We used principal component analysis (PCA-env sensu; Broennimann et al. (2012)) to reduce the dimensionality of the environmental space that was originally composed of 19 axis (bioclimatic variables). PCA-env discriminates differences between environmental backgrounds of the two species and has been shown to accurately detect niche overlaps. Therefore, this ordination technique allows for direct comparisons of species-environment relationships in environmental space, and employs various maximization criteria to construct synthetic axes from associated variables (Jongman et al. 1995).

The PCA-env was constructed based on the combination of the 19 climatic variables across the environmental background of the native biome where the two species occur. The first two PCA-env axes explained 63% of the environmental variance. This environmental space was then gridded into 100 × 100 cells bounded by minimum and maximum values present in the background data following Broennimann et al. (2012). We standardized the number of occurrences of both species at each gridded cell to avoid oversampling specific environmental conditions (abundance biases). A smoothed kernel density function with a standard bandwidth was calculated on species occurrences in each grid cell to allow the direct comparison of species densities in light of the available environment (Broennimann et al. 2012). We assumed the smoothed density of occurrences such as the probability distribution defined over the multivariate environmental space, and calculated Schoener’s *D* in this multivariate climatic space. Schoener’s *D* ranges from 0 (complete discordance) to 1 (identical niches). We tested if the observed overlap is greater than expected from a random distribution of 1000 points. Separately, we tested if Schoener’s *D* was similar when calculated using all the 19 environmental variables and when using the reduced set of climatic variables that were selected for species distribution models (see section on SDM’s for more details on variable selection). All the calculations were performed with the ecospat package (Broennimann et al. 2015) in R (Ihaka and Gentleman 1996).

### Overlap in the functional role

**Fig. 1:**
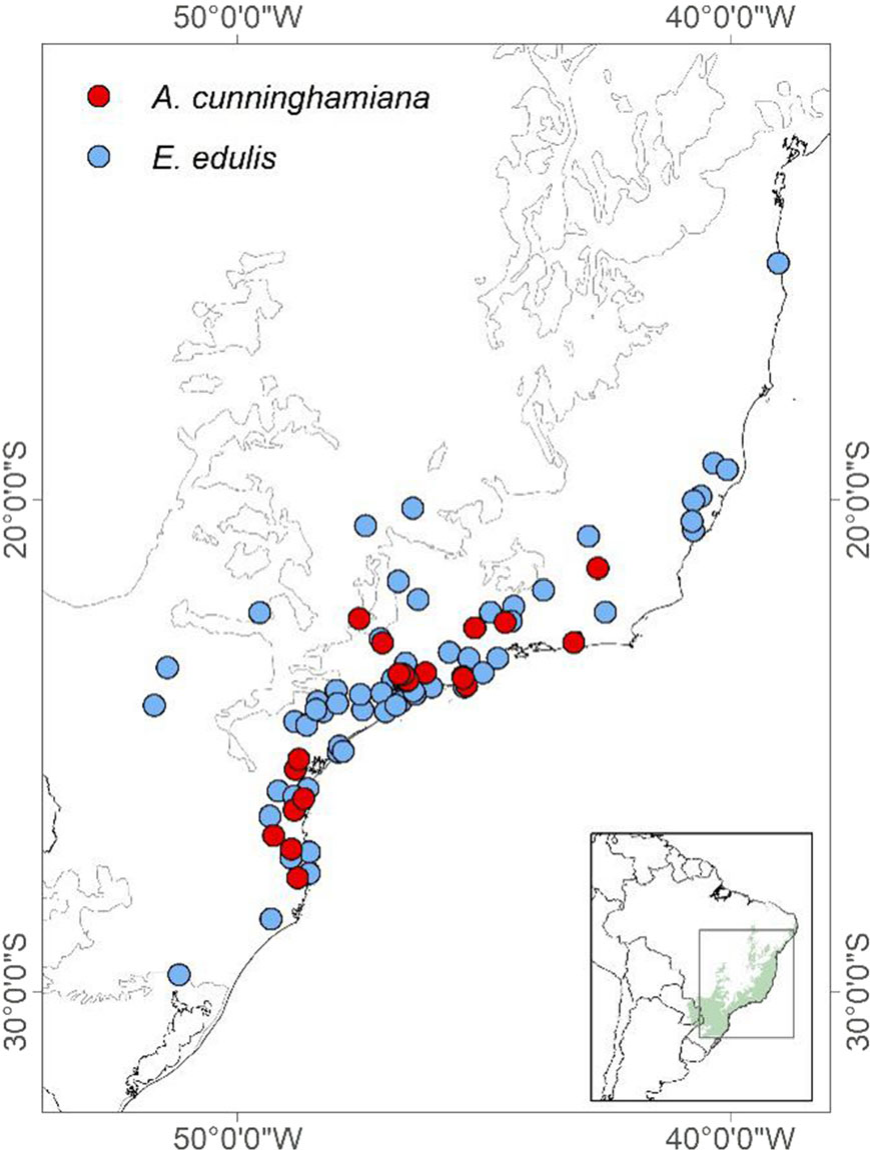
Location of the frugivory interactions reported for the two species. In blue *E. edulis* and in red *A. cunninghamiana.* The bottom right frame illustrates the location of the Atlantic Forest in Brazil

We compiled 482 frugivory interactions from the literature reported in 77 different sites across the Atlantic Forest: 92 records for *A. cunninghamiana* and 388 records for *E. edulis* (Bello et al. 2017; Castro 2003; Cintra 2016; Galetti et al. 2013; Matos and Pivello 2009) (Fig. 1, Table S2). First, we compared the set of bird species that interacts with each palm using Sørensen beta-diversity (β_SØR_), because it fulfills many of the desirable properties of a beta diversity metric, such as symmetry and homogeneity (Koleff et al. 2003). We further partitioned β_SØR_ into its turnover and nestedness components to respectively assess how much of the difference in species composition is due to species replacement and how much is due to differences in species richness (birds that interact with one palm are a subset of those that interact with the other palm).

To assess the functional role of the palms in the Atlantic Forest, we estimated the functional niche of each palm species as multidimensional trait space based on the functional traits of the interacting bird species, following Dehling et al. (2016). For the 74 bird species interacting with the two palms, we obtained trait information from Rodrigues et al. (2019). We included bird traits that are related to frugivory: body mass (g), bill width (mm), and wing length (mm). Body mass is related to competitive abilities for resources, given that larger birds are often competitively dominant over smaller birds (Feinsinger and Colwell 1978). Bill width limits the size of fruits that a bird can ingest (Galetti et al. 2013). Wing length confers the ability to access more resources due to high mobility in dense forest (Denslow and Moermond 1985). We measured functional niche overlap using the Schoener’s *D* and its significance as we did for the environmental niche (Broennimann et al. 2012, 2015; Warren et al. 2008).

### Species distribution models (SDMs)

To infer the potential distribution of the two palm species in the Atlantic Forest under current and future climatic conditions, we calibrated SDMs using species occurrences and environmental variables from the distribution range of each species in Australia and the Atlantic Forest to maximize the quality of predictions (Gallien et al. 2010). We selected the original biome distribution for each species as background, following Dinerstein et al. (2017). For *E. edulis* we selected the Atlantic Forest of Brazil as the original biome. For *A. cunninghamiana,* we selected the Queensland tropical rainforests and southeast Australian temperate forests for models built using the native range. For the global model we used all records across the globe to identify biomes where *A. cunninghamiana* is established and then used these biomes as background.

A careful selection of environmental variables is important to obtain realistic predictions of invader distributions especially when the distribution is projected into novel environments (Sheppard and Gonzalez-Andujar 2013). We used factorial analysis to reduce collinearity among variables, selecting one representative variable related with each significant factor (eigenvector), similar to the variable selection procedure adopted by Sobral-Souza et al. (2015) from the original 19 bioclimatic variables (Table S3). We identified five significant factors for *E. edulis* and six factors for *A. cunninghamiana.* If a selected variable had high correlation with another selected variable, we discarded it and selected the next variable that contributed to the factor. For *E. edulis* we selected isothermality (bio 3), temperature annual range (bio 7), mean temperature of warmest quarter (bio 10), precipitation of wettest quarter (bio 16), and precipitation of coldest quarter (bio 19). For *A. cunninghamiana* we selected isothermality (bio 3), temperature seasonality (bio4), mean temperature of driest quarter (bio 9), mean temperature of warmest quarter (bio 10) and precipitation of driest quarter (bio 17). The selected variables were not strongly correlated (Pearson’s r < 0.7; Table S3, Figures S1, S2).

We used a forecast ensemble approach (Araújo and New 2007) based on four different algorithms. The first two were presence-only methods: envelope score method—Bioclim (Nix 1986)—and distance method—Domain (Gower distance; Carpenter et al. 1993). The other two were machine-learning methods based on presence-background records: support vector machines (Tax and Duin 2004) and maximum entropy—MaxEnt (Phillips and Dudík 2008). Modeling was conducted with the R-packages “dismo” and “kernlab” (Hijmans et al. 2015; Karatzoglou and Feinerer 2010; Karatzoglou et al. 2004).

We modeled each species individually using a bootstrap partition with 75% and 25% for training and testing data, respectively. The testing data were used to calculate the true skill statistic (TSS) for each model (Allouche et al. 2006). To calculate TSS, we estimated the “maximum specificity and sensitivity” threshold values, as recommended when presence-only data are available in the niche modeling analysis (Liu et al. 2013, 2016). We randomized the data and repeated this procedure 10 times for each algorithm and for each species. Thus, we obtained 40 maps (4 algorithms × 10 times) for each species. For the ensemble process, we combined only the replicates with a TSS greater than 0.5 to ensure that only well-performing models were ensembled (Allouche et al. 2006). We standardized the cell values in each model to a range between 0 and 1 and multiplied this value with the TSS of each replicate. Thus, the cell values of the final maps correspond to the weighted mean of the replicates involved in the ensemble process.

Finally, to transform the continuous map into a binary map, we calculated four thresholds for each species at the current climate scenario: 10 percentile training presence maximum sensitivity and specificity threshold, equal sensitivity and specificity and the minimum training presence. We submitted each resulting distribution map to an expert to define the threshold that best fit the current distribution of each species (10 percentile for *E. edulis* and minimum training presence for *A. cunninghamiana*). We then reclassified the predicted probability distribution of each species into presence and absence for the area of invasion (Atlantic Forest for *A. cunninghamiana*). Using the algorithm replicates with a TSS greater than 0.5 and the selected thresholds for each species at the current climate scenario, we projected each model to the future climate scenarios (rpc45 and rcp85 for 2050 and 2070) for the entire Atlantic Forest. We performed the future ensemble with the same method as for the current conditions. With the reclassified models in the Atlantic Forest, we determined the potential area of occurrence of both species that overlapped in current and future scenarios, identifying where competitive interactions might affect *E. edulis*.

## Results

**Fig. 2:**
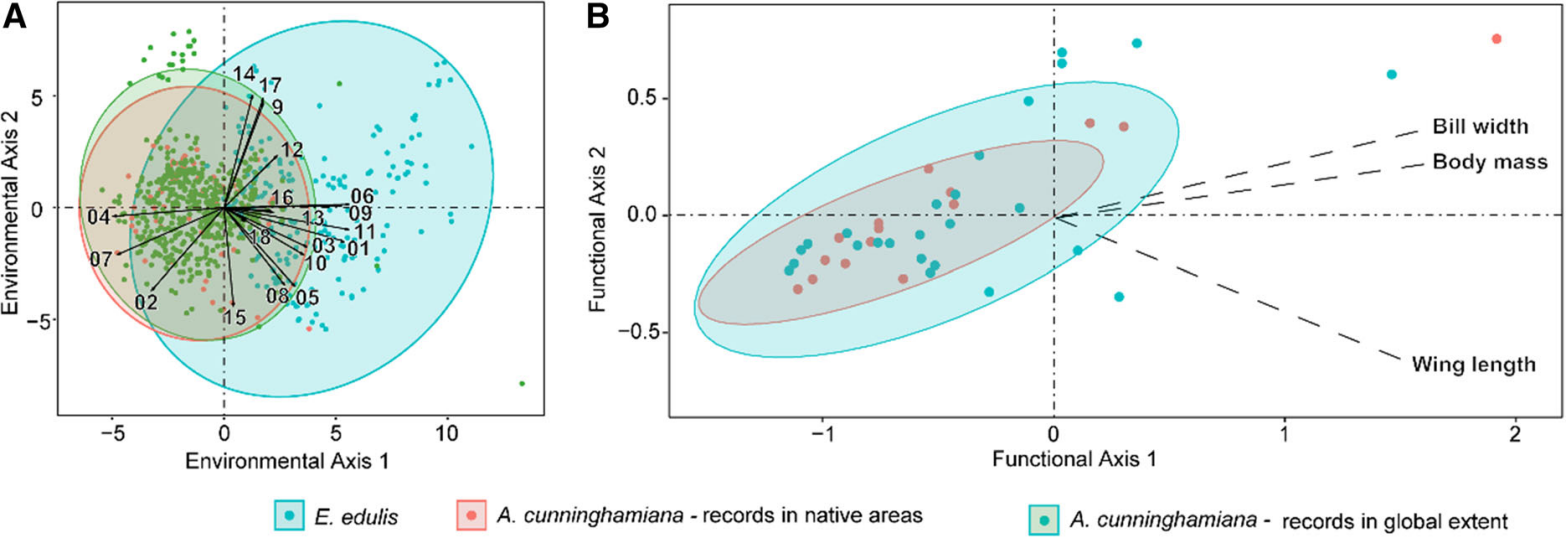
Environmental niche and functional role of *A. cunninghamiana* (red and green) and *E. edulis* (blue). **a** Environmental niche under current conditions. For *A. cunninghamiana* we present the environmental niche of the native area (red) and the environmental niche including the invaded areas on the world (green). The first environmental axis is related mostly with temperature variables and isothermality. Numbers represent the bioclimatic variables (bio1: annual mean temperature, bio3: isothermality, bio4: temperature seasonality, bio 7: temperature annual range, bio 9: mean temperature of driest quarter, bio10: mean temperature of warmest quarter and bio11: mean temperature of coldest quarter). High values of the x-axis generally correspond to warmer areas while small values correspond with seasonal temperatures. The second axis is related with precipitation variables (bio12: annual precipitation, bio14: precipitation of driest month, bio15: precipitation seasonality, bio17: precipitation of driest quarter, bio18: precipitation of warmest quarter, bio19: precipitation of coldest quarter). High values of the y-axis generally correspond to wetter areas while small values correspond with strong seasonal precipitation. **b** Functional role based on traits of the species of frugivores that interact with each palm. The first functional axis is related to body mass and bill width and the second functional axis is related to wing length. High values of the x-axis generally correspond to birds with high body mass, broad bill widths and higher values in y-axis correspond to birds with long wings. Each graph represents the first factorial plane of a PCA ordination, which explains 63% for the environmental niche and 90% of the variance of the functional role. Points represent the occurrences and circles include the 95% of occurrence. Black arrows represent the direction of variables in the first factorial plane. See Table S3 for the names of the all bioclimatic variables

We found that *A. cunninghamiana* and *E. edulis* overlap partially in their environmental niche and frugivory functional role. The native palm *E. edulis* prefers warm and wet climates with a narrower range of temperatures. In contrast, the invasive palm, *A. cunninghamiana*, endures cold seasonal climates with a wide range of temperature variation. Moreover, *A. cunninghamiana* is expanding its niche to areas with less precipitation seasonality where the precipitation in the driest and coldest quarters of the year is higher (Fig. 2a). Despite such differences, both species can survive under some similar environmental conditions, as 29% to 39% of their environmental niche overlaps (D = 0.29, p = 1, when considering just the native range of *A. cunninghamiana,* and D = 39, p = 0.5, when considering the native and invaded range of *A. cunninghamiana*, Fig. 2a). In the areas where both species can co-occur, they potentially have similar functional roles. We found 73 bird species interacting with both palms, 37 species interacting only with *A. cunninghamiana* and only 47 with *E. edulis*. The proportion of bird generalist species interacting with *A. cunninghamiana* is 65%, whereas *E. edulis* is consumed by higher proportion of bird specialist species (46%) than *A. cunninghamiana* (35%) (Table S4). The species with more interactions reported with *A. cunninghamiana* were *Ramphastos vitellinus* (Channel-billed toucan)*, R. dicolorus* (Green-billed toucan), and with *E. edulis* were *R. dicolorus* (Green-billed toucan)*, Aburria jacutinga* (Black-fronted piping guan), and *Turdus flavipes* (Yellow-legged thrush)*, T. albicollis* (White-necked thrush). The dissimilarity of shared frugivores resulted in 0.57 beta-Sørensen index (0.46 due to turnover and 0.11 due to nestedness). Despite the relatively low proportion of shared frugivores and high turnover between the two plants, we found a high overlap in the functional niche of the palms (D = 0.84, p = 0.01; Fig. 2b).

**Fig. 3:**
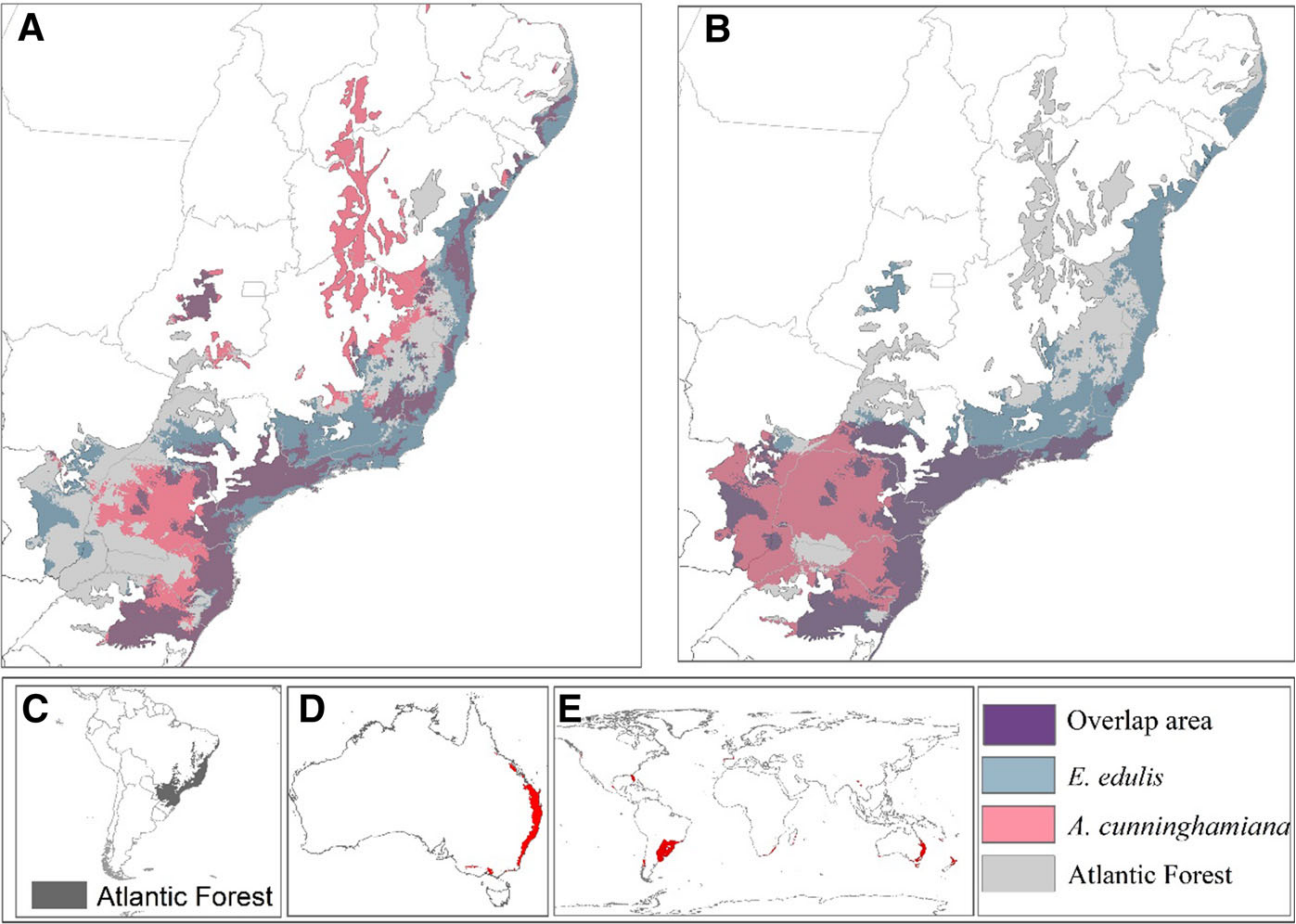
Predicted current area of distributions and overlap (purple) of *E. edulis* (blue) and *A. cunninghamiana* (red) in the Atlantic Forest (gray) under current climatic condition. **a** Shows the potential distribution and overlap between the two species when we consider just the occurrence in the native range of each species. **b** Shows the potential distribution and overlap between the two species when we consider the occurrence of *A. cunninghamiana* in its global extent. **c** Location of the Atlantic Forest in Brazil. **d** Potential distribution of *A. cunninghamiana* in its native range of distribution (Australia). **e** Potential distribution of *A. cunninghamiana* in all the world when considered all the records of the species

**Fig. 4:**
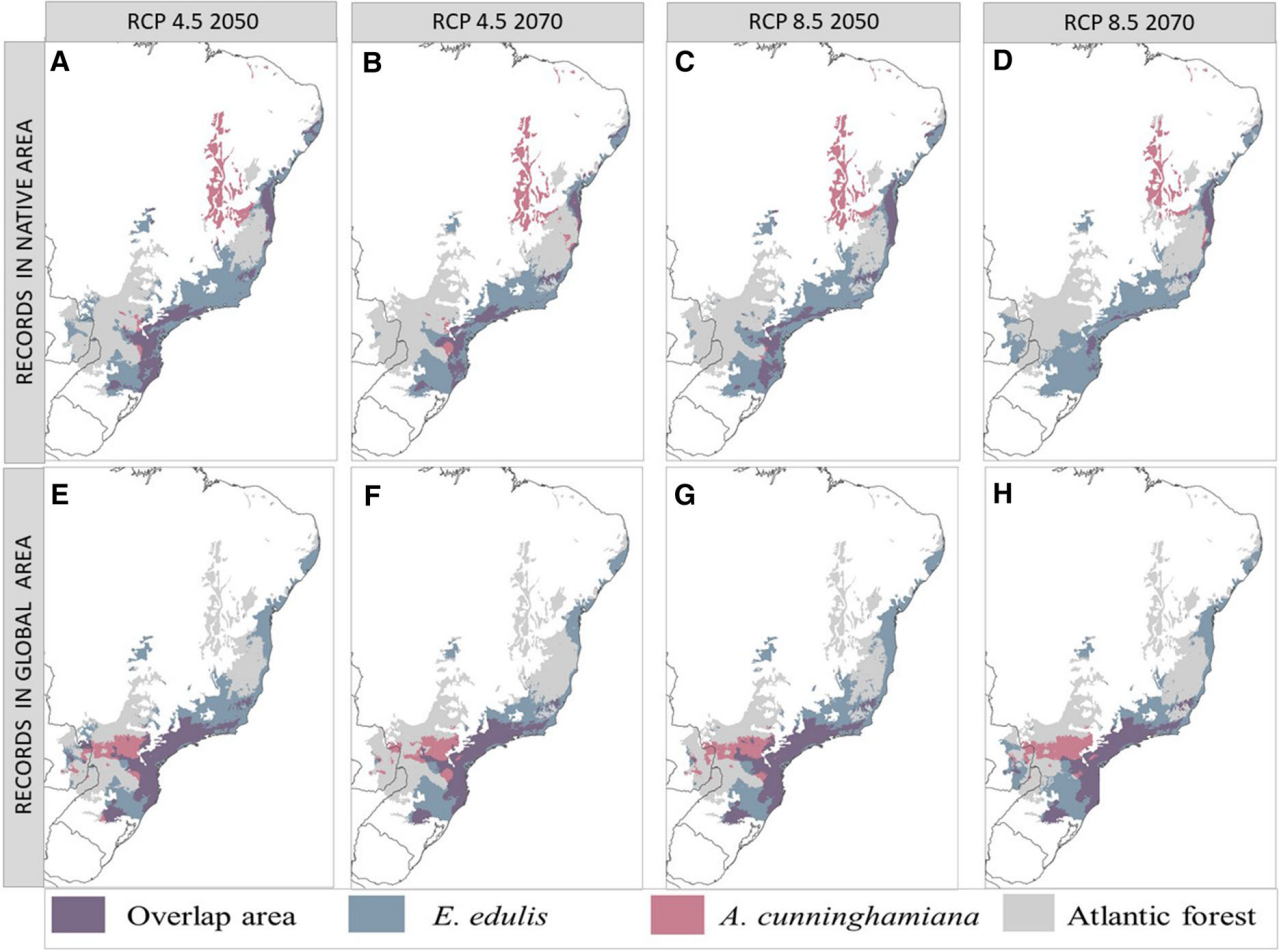
Predicted area of distributions of *E. edulis* (blue), *A. cunninghamiana* (red), and their overlap (purple) in the Atlantic Forest (grey) under four future climatic change scenarios. The first row shows the predictions if we consider only records in the native area of *A. cunninghamiana.* The second row shows the predictions if we consider the records in the global extent for *A. cunninghamiana*. Each column represents a representative concentration pathway (RCP) of climate change: **a** RCP45 for year 2050 with native records of *A. cunninghamiana*, **b** RCP 85 for year 2050 with native records of *A. cunninghamiana*, **c** RCP 45 for year 2070 with native records of *A. cunninghamiana*, and **d** RCP 85 for year 2070 with native records of *A. cunninghamiana*, **e** RCP45 for year 2050 with native and invasive records of *A. cunninghamiana*, **f** RCP 85 for year 2050 with native records and invasive of *A. cunninghamiana*, **g** RCP 45 for year 2070 with native and invasive records of *A. cunninghamiana*, and **h** RCP 85 for year 2070 with native and invasive records of *A. cunninghamiana*

The potential area of distribution of *E. edulis* covers 46% of the Atlantic Forest and *A. cunninghamiana* can occur between 44% to 82% of the Atlantic Forest (Fig. 3). However, just 50% of the distribution area of the native palm is threatened by invasion by *A. cunninghamiana* (Fig. 3, Table 1). The northwest area of the Atlantic forest (São Francisco biogeographic region) appears as a potential area of invasion when the *A. cunninghamiana* model is calibrated in the native range (Fig. 3a). However, this area presents high uncertainty in the prediction (Figure S3A). Consistently, the southeastern Atlantic Forest, the evergreen, semi-deciduous and Araucaria forests (mixed temperate), are most suitable for invasion (Figs. 3 and 4). The southeastern region corresponds to the coldest, most seasonal areas of *E. edulis* distribution. Further, the invasive palm can expand its distribution to the inner part of the Atlantic Forest, reaching the frontier with Paraguay and Argentina, under current climate conditions.

**Table 1:** Predicted distribution areas for each species and their overlap under current and future climate change scenarios

Climatic scenario	*E. edulis*	*A. cunninghamiana* (native range)			*A. cunninghamiana* (invasive range)		
	Area (km^2^)	Area (km^2^)	Area of overlap (km^2^)	Percentage *E. edulis* area that overlaps (%)	Area (km^2^)	Area of overlap (km^2^)	Percentage *E. edulis* area that overlaps
Current	158,370	151,380	80,305	50.71	282,710	81,835	51.67
RCP45 2050	165,250	49,855	48,155	29.14	157,830	64,870	39.26
RCP45 2070	135,450	45,110	33,525	24.75	148,415	56,840	41.96
RCP85 2050	160,210	49,315	37,700	23.53	157,830	64,360	40.17
RCP85 2070	154,285	32,590	15,190	9.85	157,830	60,690	39.34

The tendency for the Atlantic Forest to become warmer with high climate seasonality in the future may reduce the potential expansion of *A. cunninghamiana* into new areas and reduces its geographic overlap with the native species (Table 1 and Fig. 4). All predictions for future climatic scenarios point to a reduction in the distribution of the invasive palm especially given the decrease in the area potentially suitable for invasion in the west part of the Atlantic Forest. This reduction is less drastic if we consider that *A. cunninghamiana* can invade areas with climates different from its native range, in which case *A. cunninghamiana* can still remain in almost 50% of the Atlantic Forest (Fig. 4e–h, Table 1). In contrast, the full extent of the native palm distribution appears to change little under future climate change scenarios (Table 1).

## Discussion

The invasive palm, *Archontophoenix cunninghamiana*, has the potential to invade half of the area of distribution of the native palm *Euterpe edulis* in the Atlantic Forest and may disrupt the frugivory interactions given the overlap in the environmental niche and the functional roles of these two palm species. Despite *A. cunninghamiana* preferring colder seasonal climates and *E. edulis* preferring warmer climates, the environmental niche of the palms overlap up to 39%. This translates into *A. cunninghamiana* potentially invading nearly 50% of the current projected geographic range of *E. edulis*, especially in the seasonal and subtropical climate conditions present in the southeastern Atlantic Forest (Joly et al. 2014). Nevertheless, climate change may help to slow the invasion process by reducing the geographic area of suitable climate for *A. cunninghamiana*, reducing the area of overlap between the two species.

The strength of the invasion may be influenced by the ability of the invasive species to adapt to new environmental conditions in the invaded area (Broennimann et al. 2007). *Archontophoenix cunninghamiana* has the potential to expand its environmental niche into areas with less seasonal precipitation. This expansion suggests that invasive species can expand their niche quickly and occupy different environmental conditions in invaded areas (Broennimann et al. 2007; Ellers et al. 2011; Frost et al. 2019). Nonetheless, areas that are predicted as areas of potential invasion but have high uncertainty in the prediction, such as the northwest areas of the Atlantic forest (São Francisco biogeographical region), should be interpreted with caution (Pearson et al. 2006). This high uncertainty is related to the fact that the environmental conditions of São Francisco are not present in the native area of distribution of *A. cunninghamiana* (Figure S3B). Therefore the models do not have information to produce an accurate prediction for these conditions (Elith et al. 2010; Jiménez-Valverde et al. 2011a, b). In fact, if we consider the global model where *A. cunninghamiana* occurs in climates outside its native environmental conditions, the models improve their performance, and the current potential invasion becomes more threatening, expanding from 43 to 82% of the Atlantic Forest. Even considering this environmental flexibility, the area of invasion is predicted to decline in the future because warmer climatic conditions restrict the potential invasion area. However, the ability to support new environmental conditions attenuates this reduction allowing the invasive species to remain in almost 50% of the Atlantic Forest and affects nearly 40% of the area of distribution of *E. edulis* under climate change scenarios. A similar reduction in invasion risk under climate change has also been reported for other invasive plant species in the tropics and subtropics due to the drastic increase in temperature over the tolerance range of the invasive species (Peterson et al. 2008). Much of Central America and the South Atlantic Forest were identified as areas that could be affected at a lower rate of invasion under climate change scenarios due to the increase of temperature above the tolerance of most invasive species (Bellard et al. 2014). However, we should still pay special attention to regions that remain suitable for *A. cunninghamiana,* under current and future climate change scenarios, and especially, we should consider that the invasive species might be more threatening given its capacity adapt to new environmental conditions.

In the current or future areas where the two species potentially overlap, the invasion success is also influenced by the invader’s ability to interact with mutualistic species (Frost et al. 2019). The invasive palm can perform a similar function to that of the native palm in the communities because it interacts with a similar frugivores. Therefore, the invader has the potential to disrupt the interaction network of the native species by changing the identity (rewiring) and frequency of interaction between the frugivores and the native *E. edulis*. Generalist plant species, such as *A. cunninghamiana*, have the potential to take interactions from more-specialized native species (Aizen et al. 2008; Stouffer et al. 2014), such that pollen-transport and seed-dispersal networks can be dominated by invader’s pollen (Emer et al. 2015) or seeds (Heleno et al. 2013). In this case, *A. cunninghamiana* could compete for seed dispersers, which could result in a fitness reduction in the native species (Galetti et al. 1999; Reis et al. 2000; Williams 2008). Moreover, the prolonged fruiting period and greater fruit yield of *A. cunninghamiana* (Mengardo and Pivello 2012) may increase its number and frequency of seed-dispersal interactions (Gonzalez-Castro et al. 2012) displacing the frugivory interactions from the native *E. edulis*. Besides, *A. cunninghamiana* has higher germination and survival rates than *E. edulis* (da Luz et al. 2017; Mengardo and Pivello 2012; Mengardo and Pivello 2014). In fact, in invaded fragments, populations of *A. cunninghamiana* have higher recruitment rates (19.7% per year; Dislich et al. 2002) than that reported for most tree species in tropical forests (Melo et al. 2000). Moreover, *A. cunninghamiana* may have an advantage over *E. edulis*, given that it does not co-occur with a specific seed predator known to control its population growth (Christianini 2006; Keane and Crawley 2002).

Invasive species can also positively influence native species with similar roles (Frost et al. 2019). For example, the prolonged period of fruiting and higher fruit yield of *A. cunninghamiana* (Mengardo and Pivello 2012) may attract frugivores from surrounding areas increasing local population, such that native species may benefit from being in the vicinity of the invader (Frost et al. 2019). This effect may be important for *E. edulis* populations that persist in defaunated forests (Galetti et al. 2013). Yet, this benefit is likely to be realized only if the frugivores use both palms and do not specialize on the invader (Brosi 2016). Generalist invasive species may also alter the vulnerability of the systems to perturbations by acting as central species connecting many species, whereas natives remain peripheral. This change in network structure results in large groups of species (modules) and increased nestedness (i.e., marginal species interacting with generalist species) (Albrecht et al. 2014; Bartomeus et al. 2008; Santos et al. 2012; Stouffer et al. 2014) properties that confer less vulnerability to perturbations (Bascompte et al. 2003; Bastolla et al. 2009).

Anthropogenic factors such as fragmentation, overexploitation of *E. edulis* palm heart and climate change may influence the invasion success of *A. cunninghamiana* in different ways. Fragmentation and overexploitation of *E. edulis* can enhance the invasion of *A. cunninghamiana*, as smaller fragments are more vulnerable to biological invasions than larger areas (Laurance and Bierregaard 1997). Besides, forest fragments where *E. edulis* is now extinct or has small populations because of intensive harvesting, may still harbor a frugivore assemblage that is capable of dispersing *A. cunninghamiana* seeds and facilitating its establishment (Mengardo and Pivello 2014). Moreover, high densities of the invasive plant in fragments can act synergistically with the frugivores by increasing the presence and abundance of generalist birds (Campagnoli and Antunes 2017). We found that *A. cunninghamiana* interacts with a higher proportion of generalist than *E. edulis*. These generalist birds, which tend to use a variety of habitats, can disperse seeds from areas where *A. cunninghamiana* is cultivated for ornamental or palm heart source into forest fragments (Dislich et al. 2002).

The observed turnover between the frugivores that interact with the two species could result from the geographic distribution of frugivores and their spatial and temporal overlap with both palms (Dittel et al. 2019), or because the studies recording interactions with *A. cunninghamiana* are limited in comparison with those recorded for *E. edulis*. The morphological plant traits (small fruit size, high fecundity and long phenology) of *A. cunninghamiana* suggest that this species will interact with more species than the ones that were recorded in the present study. The broader set interactions, and new potential interactions under climate change, could influence the predicted distribution of the invasive palm in the Atlantic Forest by modifying patterns of seed-dispersal. The distribution of mutualistic frugivores will also change under climate change which may impact the potential movement of seeds into environmental suitable areas for the invasive palm (Ellers et al. 2011; Schleuning et al. 2020). Therefore, we suggest further studies on frugivore interactions with the invasive palm, especially by assessing competition in areas where the two species co-occur, and by exploring the possible asynchronism between the potential distribution of the plants and frugivores under climate change.

Finally, the expansion of the *A. cunninghamiana* palm heart industry in the southern Atlantic Forest (e.g., Santa Catarina and Rio Grande do Sul states; Corso 2013), where environmental conditions remain suitable in the future, may promote the invasion of fragments close to the plantations (Matos and Pivello 2009) and should be closely monitored. We suggest the removal of established individuals as ways to mitigate impacts and control the invasion of *A. cunninghamiana* in Brazil, in accordance with the national strategy on invasive alien species of the federal government. In practice, we suggest (1) the regular removal of inflorescences or the use of anti-bird nets in areas where *A. cunninghamiana* is cultivated for extraction of palm heart, in order to maintain this legal trade that potentially decreases the pressure of illegal extraction of the native palm, (2) the regular removal of inflorescences or the replacement of stablished individuals of *A. cunninghamiana* used for landscaping, such as what has been done in large public parks in the city of São Paulo, (3) prohibition of the use of *A. cunninghamiana* in new landscaping projects, suggesting instead the use of native *E. edulis*, whose landscape potential is similar (Sartorelli et al. 2018), (4) restoration of native areas in which the invasive palm is found in high density, in order to facilitate the reintroduction of the native palm (Christianini 2006), and (5) greater surveillance over the extraction and illegal commercialization of the native palm.

In conclusion, we found that *A. cunninghamiana* has the potential to invade half of the natural area of distribution of the keystone species *E. edulis* of the Atlantic Forest. Furthermore, *A. cunninghamiana* has the potential to occur in different environmental conditions from those present in its native range, which can facilitate the expansion in the areas of invasion, potentially impacting about 80% of the Atlantic Forest. Fortunately, climate change may help to counter this expansion because the drastic increase in temperature is predicted to limit the expansion of *A. cunninghamiana*. However, in the areas where both species are predicted to co-occur, *A. cunninghamiana* has the potential to disrupt the frugivory interactions of the native palm, given their similarity in functional roles. With the analyses of potential invasion of *A. cunninghamiana* in the Atlantic Forest, we highlighted that current and future environmental requirements within the native area and in the areas where invasion has occurred, as well as the functional roles of the invasive species are important to consider in the analysis of biological invasion risk.

## Electronic supplementary material

Below is the link to the electronic supplementary material.

Supplementary material 1 (DOCX 1139 kb)

Supplementary material 2 (XLSX 22 kb)

Supplementary material 3 (XLSX 31 kb)
