# Supplementary material for: Environmental niche and functional role similarity between invasive and native palms in the Atlantic Forest
Source: Biol Invasions. 2020 Nov 13;23(3):741–54. doi: 10.1007/s10530-020-02400-8 (PMC7900028; doi:10.1007/s10530-020-02400-8)
Supplement: Supplementary file 1 — Supplementary material 1 (DOCX 1139 kb) [file 10530_2020_2400_MOESM1_ESM.docx]

**Environmental and functional niche similarity between invasive and native palms in the Brazilian Atlantic Forest**

Carolina Bello^1,2*^, Ana Laura P. Cintra^1^, Elisa Barreto^2,3^, Maurício Humberto Vancine^1^, Thadeu Sobral-Souza^1,4^, Catherine H. Graham^2^, Mauro Galetti^1^

**Supplementary Information:**

**Table S1. List of 159 occurrence records used to model *A. cunninghamiana* and 248 ocurrence records for *E. edulis.*** Data are available in the file Supplemental table 1.xls

**Table S2**. **List of frugivory registered for *A. cunninghamiana and E. edulis* along the Atlantic forest.** The data show pairwise interactions of palms and animals (palm species, animal), the reference of the study from which the information was extracted (reference) and the location with municipality (municipality) and coordinates (latitude and longitude). Data are available in the file supplemental table 2.xls

**Table S3. Variable contribution to each factor (F) and proportion of variance explain for each palm species.** In bold are the selected variables for modelling the distribution of each species.

|  |  | ***E. edulis*** | | | | | ***A. cunninghamiana*** | | | | | |
| --- | --- | --- | --- | --- | --- | --- | --- | --- | --- | --- | --- | --- |
| Proportion of variance explain | | F1 | F4 | F2 | F3 | F5 | F1 | F5 | F3 | F2 | F4 | F6 |
|  |  | 0.29 | 0.28 | 0.15 | 0.14 | 0.09 | 0.27 | 0.23 | 0.19 | 0.15 | 0.07 | 0.05 |
| bio01 | Annual Mean Temperature | 0.9 | 0.32 | 0.07 | 0.18 | 0.23 | 0.39 | 0.86 | 0.15 | 0.27 | 0.04 | 0.12 |
| bio02 | Mean Diurnal Range | 0 | 0.23 | 0.07 | 0.96 | 0.11 | 0.25 | 0.06 | 0.93 | 0.15 | 0.15 | 0.14 |
| **bio03** | **Isothermality** | 0.28 | 0.63 | 0 | 0.04 | **0.62** | 0.44 | 0.21 | 0.28 | 0.24 | 0.16 | **0.77** |
| **bio04** | **Temperature Seasonality** | 0.28 | 0.61 | 0.03 | 0.44 | 0.56 | 0.37 | 0.01 | **0.86** | 0.03 | 0.02 | 0.34 |
| bio05 | Max Temperature of Warmest Month | 0.92 | 0.09 | 0.15 | 0.28 | 0.09 | 0.06 | 0.76 | 0.5 | 0.33 | 0.22 | 0.07 |
| bio06 | Min Temperature of Coldest Month | 0.72 | 0.23 | 0.13 | 0.55 | 0.31 | 0.42 | 0.62 | 0.57 | 0.21 | 0.24 | 0.1 |
| **bio07** | **Temperature Annual Range** | 0.17 | 0.22 | 0.04 | **0.91** | 0.31 | 0.35 | 0.01 | 0.92 | 0.06 | 0.05 | 0.15 |
| bio08 | Mean Temperature of Wettest Quarter | 0.77 | 0.38 | 0.01 | 0.06 | 0.09 | 0.32 | 0.73 | 0.04 | 0.14 | 0.54 | 0.17 |
| **bio09** | **Mean Temperature of Driest Quarter** | 0.73 | 0.2 | 0.08 | 0.3 | 0.48 | 0.03 | 0.12 | 0.14 | 0.15 | **0.85** | 0.05 |
| **bio10** | **Mean Temperature of Warmest Quarter** | **0.99** | 0.09 | 0.11 | 0.05 | 0.01 | 0.26 | **0.91** | 0.16 | 0.27 | 0.07 | 0.01 |
| bio11 | Mean Temperature of Coldest Quarter | 0.79 | 0.41 | 0.05 | 0.29 | 0.35 | 0.44 | 0.75 | 0.37 | 0.24 | 0.07 | 0.19 |
| bio12 | Annual Precipitation | 0.25 | 0.76 | 0.56 | 0.1 | 0.14 | 0.87 | 0.09 | 0.3 | 0.37 | 0.05 | 0.06 |
| bio13 | Precipitation of Wettest Month | 0.07 | 0.16 | 0.96 | 0.03 | 0.04 | 0.93 | 0.21 | 0.26 | 0.08 | 0.04 | 0.08 |
| bio14 | Precipitation of Driest Month | 0.28 | 0.92 | 0.14 | 0.06 | 0.16 | 0.06 | 0.33 | 0.03 | 0.88 | 0.15 | 0.07 |
| bio15 | Precipitation Seasonality | 0.2 | 0.85 | 0.33 | 0.02 | 0.27 | 0.76 | 0.32 | 0.23 | 0.39 | 0 | 0.19 |
| **bio16** | **Precipitation of Wettest Quarter** | 0.07 | 0.16 | **0.98** | 0.01 | 0.02 | **0.93** | 0.2 | 0.27 | 0.08 | 0.06 | 0.09 |
| **bio17** | **Precipitation of Driest Quarter** | 0.27 | 0.93 | 0.13 | 0.09 | 0.16 | 0.09 | 0.25 | 0.06 | **0.96** | 0.09 | 0.07 |
| bio18 | Precipitation of Warmest Quarter | 0.38 | 0.09 | 0.6 | 0.19 | 0.45 | 0.85 | 0.39 | 0.21 | 0.04 | 0.18 | 0.12 |
| **bio19** | **Precipitation of Coldest Quarter** | 0.11 | **0.9** | 0.02 | 0.13 | 0.1 | 0.14 | 0.5 | 0.2 | 0.55 | 0.35 | 0.15 |

**Table S4. Summary of frugivory registered for *A. cunninghamiana and E. edulis* and their traits.** We present the number of register found for each interaction, the body mass (g), the bill with (mm), the wing length (mm) and the level of specialization

| Palm | Birds | Number of registers | Body mass | Wing length | Bill width | Level specialization |
| --- | --- | --- | --- | --- | --- | --- |
| *A. cunninghamiana* | *Aratinga leucophtalmus* | 1 | NA | NA | NA | specialist |
|  | *Baryphthengus ruficapillus* | 1 | 12.2 | 130.85 | 150 | generalist |
|  | *Brotogeris chiriri* | 1 | NA | 61.67 | 112.1 | specialist |
|  | *Brotogeris tirica* | 1 | 9.86 | 67.75 | 114.06 | specialist |
|  | *Euphonia violacea* | 1 | 7.61 | 15.1 | 59.04 | specialist |
|  | *Forpus xanthopterygius* | 1 | 8.96 | 25.06 | 84.49 | specialist |
|  | *Megarynchus pitangua* | 1 | 17.1 | 57.22 | 109.14 | generalist |
|  | *Mimus saturninus* | 3 | 9.16 | 72.26 | 113.95 | generalist |
|  | *Myiodynastes maculatus* | 1 | 12.22 | 45.25 | 106.49 | generalist |
|  | *Ortalis guttata* | 1 | NA | NA | NA | generalist |
|  | *Passer domesticus* | 1 | 8.66 | 24.45 | 75.12 | generalist |
|  | *Penelope obscura* | 2 | 21.04 | 1284.24 | 314.86 | specialist |
|  | *Penelope superciliaris* | 1 | NA | 741 | NA | specialist |
|  | *Piaya cayana* | 1 | 16.83 | 104.52 | 173.36 | generalist |
|  | *Pionus maximiliani* | 1 | 17.25 | 256 | 181.17 | specialist |
|  | *Pitangus sulphuratus* | 3 | 13.21 | 61.73 | 118.33 | generalist |
|  | *Pteroglossus aracari* | 1 | 28.46 | 237.5 | NA | specialist |
|  | *Pyroderus scutatus* | 2 | 24.07 | 303 | 270 | specialist |
|  | *Ramphastos dicolorus* | 22 | 31.44 | 350.84 | 196 | specialist |
|  | *Ramphastos toco* | 2 | 39.98 | 650 | 254 | specialist |
|  | *Ramphastos vitellinus* | 15 | 30.22 | 309.4 | NA | specialist |
|  | *Ramphocelus carbo* | 1 | 12.51 | 27.76 | 81.76 | generalist |
|  | *Salvator merianae* | 1 | NA | NA | NA | generalist |
|  | *Tangara cayana* | 1 | 7.9 | 19.44 | 72.75 | generalist |
|  | *Tangara cyanoptera* | 1 | 9.7 | 39.19 | 93.59 | generalist |
|  | *Tangara ornata* | 1 | 9.02 | 41.28 | 88.43 | generalist |
|  | *Tangara palmarum* | 5 | 9.62 | 37.52 | 93.22 | generalist |
|  | *Tangara sayaca* | 2 | 8.02 | 33.97 | 90.44 | generalist |
|  | *Tangara seledon* | 1 | 6.75 | 18.42 | 68.36 | generalist |
|  | *Thraupis episcopus* | 1 | NA | NA | NA | generalist |
|  | *Turdus amaurochalinus* | 1 | 8.6 | 59.67 | 112.82 | generalist |
|  | *Turdus flavipes* | 2 | 11.81 | 60.37 | 112.03 | generalist |
|  | *Turdus leucomelas* | 3 | 11 | 63.41 | 114.8 | generalist |
|  | *Turdus rufiventris* | 5 | 9.44 | 72.42 | 118.8 | generalist |
|  | *Tyrannus melancholicus* | 1 | 12.08 | 39.55 | 109.3 | generalist |
|  | *Tyrannus savana* | 2 | 6.89 | 30.71 | 108.48 | generalist |
|  | *Zonotrichia capensis* | 1 | 6.49 | 21.11 | 67.15 | generalist |
| *E. edulis* | *Aburria jacutinga* | 36 | NA | NA | NA | specialist |
|  | *Aramides cajanea* | 1 | NA | NA | NA | generalist |
|  | *Baillonius bailloni* | 11 | NA | NA | NA | specialist |
|  | *Baryphthengus ruficapillus* | 7 | 12.2 | 130.85 | 150 | generalist |
|  | *Brotogeris tirica* | 1 | 9.86 | 67.75 | 114.06 | specialist |
|  | *Cacicus haemorrhous* | 1 | 10.29 | 70.72 | 138.1 | generalist |
|  | *Carpornis cucullata* | 4 | 12.95 | 80.45 | 121.29 | specialist |
|  | *Carpornis melanocephala* | 2 | 15.82 | 65.67 | 100.15 | specialist |
|  | *Celeus flavescens* | 3 | 12.11 | 134.03 | 157.32 | generalist |
|  | *Crax blumenbachii* | 1 | NA | NA | NA | specialist |
|  | *Cyanocorax caeruleus* | 3 | 15.66 | 298.67 | 203.67 | generalist |
|  | *Euphonia pectoralis* | 1 | 7.91 | 15.67 | 62.95 | specialist |
|  | *Euphonia violacea* | 1 | 7.61 | 15.1 | 59.04 | specialist |
|  | *Forpus xanthopterygius* | 1 | 8.96 | 25.06 | 84.49 | specialist |
|  | *Lipaugus lanioides* | 7 | 11.5 | 88.83 | NA | specialist |
|  | *Lipaugus vociferans* | 1 | NA | 36 | NA | specialist |
|  | *Mimus saturninus* | 3 | 9.16 | 72.26 | 113.95 | generalist |
|  | *Myiodynastes maculatus* | 3 | 12.22 | 45.25 | 106.49 | generalist |
|  | *Orthogonys chloricterus* | 2 | 9.55 | 38.1 | 96.1 | specialist |
|  | *Oxyruncus cristatus* | 1 | 9.12 | 44.23 | 92.5 | generalist |
|  | *Penelope obscura* | 7 | 21.04 | 1284.24 | 314.86 | specialist |
|  | *Penelope superciliaris* | 2 | NA | 741 | NA | specialist |
|  | *Pitangus sulphuratus* | 5 | 13.21 | 61.73 | 118.33 | generalist |
|  | *Procnias nudicollis* | 14 | 18.45 | 132.67 | 153 | specialist |
|  | *Pteroglossus aracari* | 7 | 28.46 | 237.5 | NA | specialist |
|  | *Pyroderus scutatus* | 21 | 24.07 | 303 | 270 | specialist |
|  | *Ramphastos dicolorus* | 56 | 31.44 | 350.84 | 196 | specialist |
|  | *Ramphastos toco* | 4 | 39.98 | 650 | 254 | specialist |
|  | *Ramphastos vitellinus* | 25 | 30.22 | 309.4 | NA | specialist |
|  | *Saltator similis* | 1 | 11.47 | 46.81 | 98.71 | generalist |
|  | *Salvator merianae* | 1 | NA | NA | NA | generalist |
|  | *Selenidera maculirostris* | 17 | 23.68 | 163.13 | 127.09 | specialist |
|  | *Tachyphonus coronatus* | 3 | 8.9 | 27.51 | 81.49 | generalist |
|  | *Tangara cyanocephala* | 1 | 7.09 | 15.97 | 65.5 | generalist |
|  | *Tangara seledon* | 3 | 6.75 | 18.42 | 68.36 | generalist |
|  | *Tijuca atra* | 1 | NA | NA | NA | specialist |
|  | *Tityra cayana* | 4 | 14.66 | 71.3 | 124 | generalist |
|  | *Trogon rufus* | 2 | 17.9 | 61.05 | 122.47 | generalist |
|  | *Trogon surrucura* | 2 | 17.55 | 65.5 | 141.92 | generalist |
|  | *Trogon viridis* | 20 | 17.73 | 103.83 | NA | generalist |
|  | *Trogon viridis* | 1 | NA | NA | NA | generalist |
|  | *Turdus albicollis* | 25 | 9.37 | 66.78 | 112.45 | generalist |
|  | *Turdus amaurochalinus* | 7 | 8.6 | 59.67 | 112.82 | generalist |
|  | *Turdus flavipes* | 32 | 11.81 | 60.37 | 112.03 | generalist |
|  | *Turdus leucomelas* | 8 | 11 | 63.41 | 114.8 | generalist |
|  | *Turdus rufiventris* | 14 | 9.44 | 72.42 | 118.8 | generalist |
|  | *Turdus subalaris* | 1 | NA | NA | NA | generalist |

**Table S5. Summary of the frugivory traits interacting with each species.** We present the number of species, the body mass (g), the bill with (mm) and the Wing length (mm). In parenthesis the range of the variable

|  | N. of species | Body mass | Bill length | Wing length |
| --- | --- | --- | --- | --- |
| *A. cunninghamiana* | 37 | 162.5 | 33.5 | 125.5 |
|  |  | (15.1 -1284.2) | (9.72 - 197.3) | (59 -314.8) |
| *E. edulis* | 57 | 158.9 | 32.7 | 130.5 |
|  |  | (15.1 -1284.2) | (9.72 - 197.3) | (59 -314.8) |


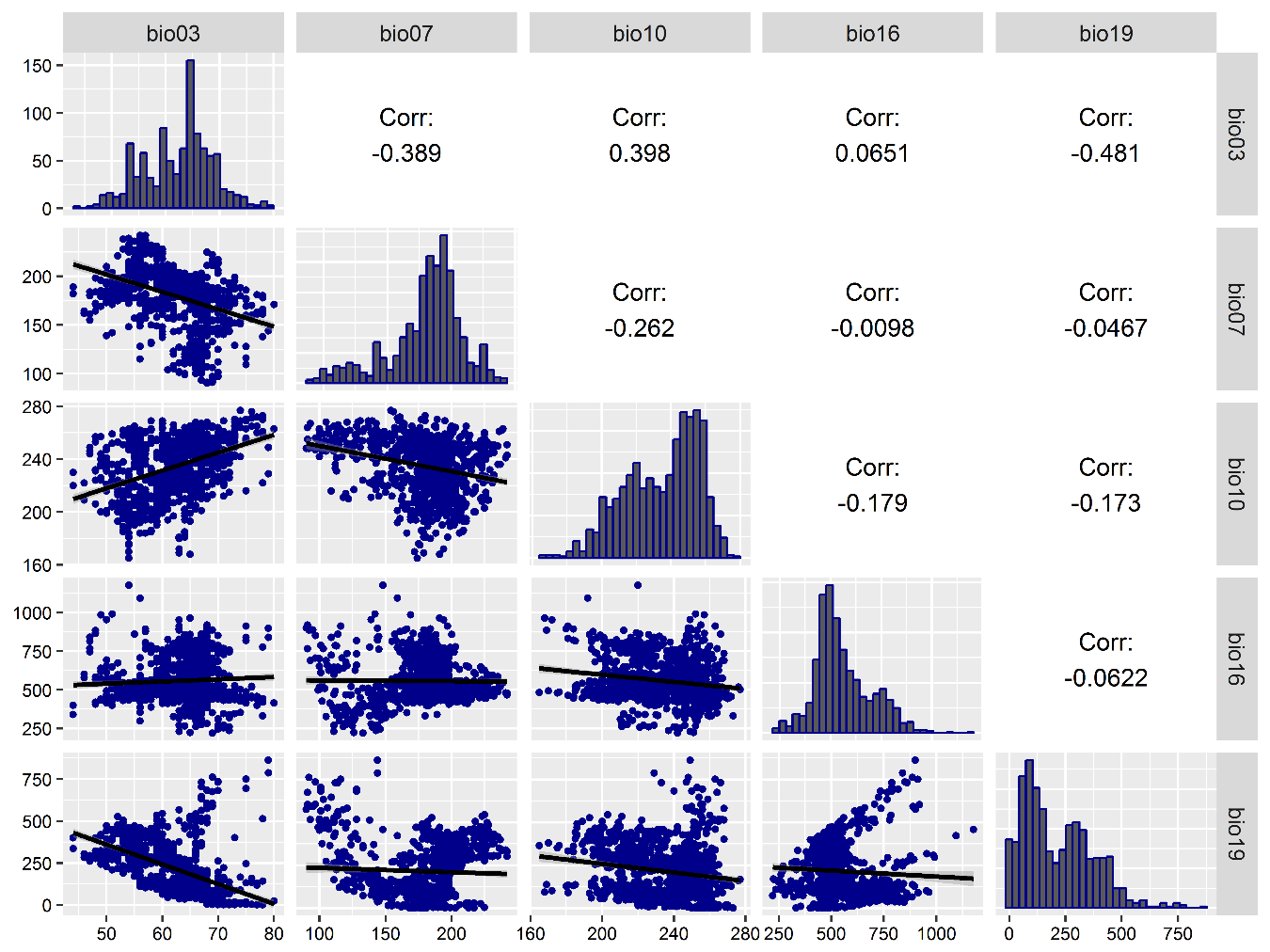


**Figure S1. Correlation between the selected variables to model *E. edulis*.** The upper diagonal reports correlation Pearson coefficient. In lower diagonal the dispersion plot of the two variables in blue and the linear tendency in black and shown. In the diagonal is the distribution of each variable.


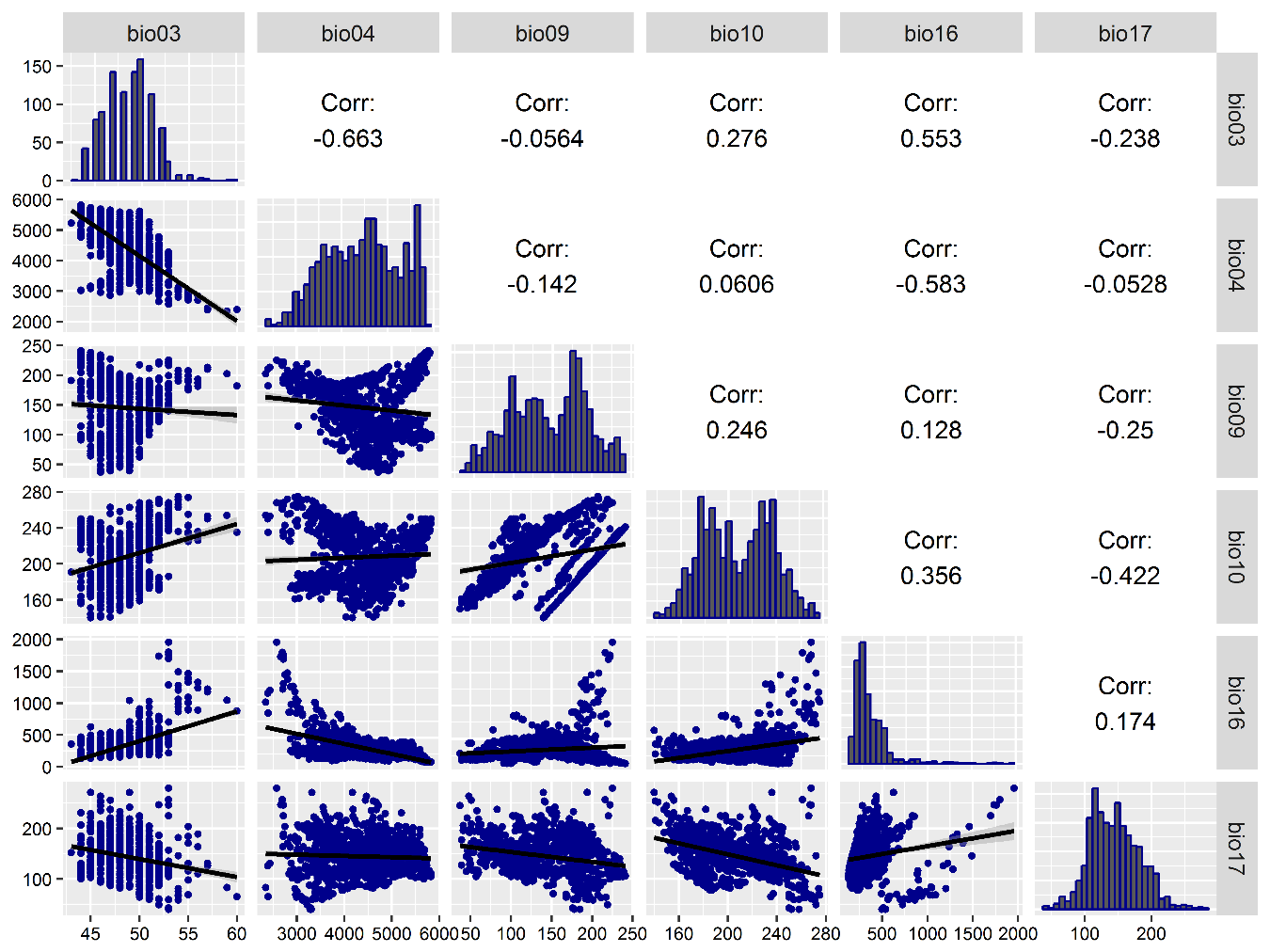


**Figure S2. Correlation between the selected variables to model *A. cunninghamiana.*** The upper diagonal reports correlation Pearson coefficient. In lower diagonal, the dispersion plot of the two variables in blue and the linear tendency in black and shown. In the diagonal is the distribution of each variable.


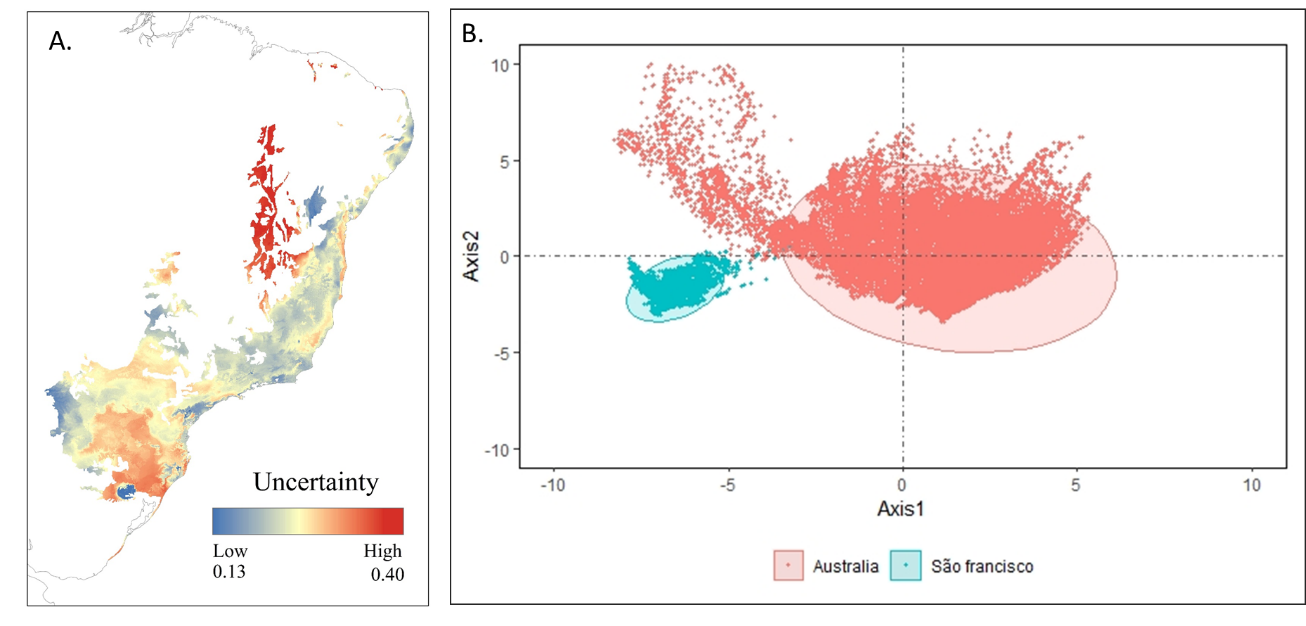


**Figure S3. Uncertainty of model ensembles for *A. cunninghamiana* when calibrating the models in the native area of distribution and projecting to the Atlantic Forest. (A)** Shows the level of uncertainty (standard deviation sd) of the *A. cunninghamiana* ensemble when the models were calibrated for the native area of distribution in Australia and projected to the Atlantic Forest. **(B)** Shows the principal coordinate analysis (PCA) of the environmental conditions present in the native area of distribution of *A. cunninghamiana* (Australia in red) and the environmental conditions in the area of São Francisco (blue), which have high uncertainty. Points represent each grid value in Australia and São Francisco and ellipses group 95% of the points.
